# Optimizing appointment template and number of staff of an OB/GYN clinic – micro and macro simulation analyses

**DOI:** 10.1186/s12913-015-1007-9

**Published:** 2015-09-16

**Authors:** R.B. Lenin, Curtis L. Lowery, Wilbur C. Hitt, Nirvana A. Manning, Peter Lowery, Hari Eswaran

**Affiliations:** 1 Department of Mathematics, University of Central Arkansas, 201 Donaghey Avenue, Conway, 72035 Arkansas USA; 2 Department of OB/GYN, University of Arkansas for Medical Sciences, 4301 West Markham Street, Little Rock, 72205 Arkansas USA; 3 College of Medicine, University of Arkansas for Medical Sciences, 4301 West Markham Street, Little Rock, 72205 Arkansas USA

**Keywords:** Outpatients, Patient types, Patient tracker data, Appointment template, Exam rooms, Optimization, Utilization, Simulation

## Abstract

**Background:**

The Department of Obstetrics and Gynecology (OB/GYN) at the University of Arkansas for Medical Sciences (UAMS) tested various, new system-restructuring ideas such as varying number of different types of nurses to reduce patient wait times for its outpatient clinic, often with little or no effect on waiting time. Witnessing little progress despite these time-intensive interventions, we sought an alternative way to intervene the clinic without affecting the normal clinic operations.

**Aim:**

The aim is to identify the optimal (1) time duration between appointments and (2) number of nurses to reduce wait time of patients in the clinic.

**Methods:**

We developed a discrete-event computer simulation model for the OB/GYN clinic. By using the patient tracker (PT) data, appropriate probability distributions of service times of staff were fitted to model different variability in staff service times. These distributions were used to fine-tune the simulation model. We then validated the model by comparing the simulated wait times with the actual wait times calculated from the PT data. The validated model was then used to carry out “what-if” analyses.

**Results:**

The best scenario yielded 16 min between morning appointments, 19 min between afternoon appointments, and addition of one medical assistant. Besides removing all peak wait times and bottlenecks around noon and late in the afternoon, the best scenario yielded 39.84 % (*p*<.001), 30.31 % (*p*<.001), and 15.12 % (*p*<.001) improvement in patients’ average wait times for providers in the exam rooms, average total wait time at various locations and average total spent time in the clinic, respectively. This is achieved without any compromise in the utilization of the staff and in serving all patients by 5pm.

**Conclusions:**

A discrete-event simulation model is developed, validated, and used to carry out “what-if” scenarios to identify the optimal time between appointments and number of nurses. Using the model, we achieved a significant improvement in wait time of patients in the clinic, which the clinic management initially had difficulty achieving through manual interventions. The model provides a tool for the clinic management to test new ideas to improve the performance of other UAMS OB/GYN clinics.

## Background

Healthcare delivery is becoming increasingly complicated and difficult to provide efficiently. Patient satisfaction is a powerful force in the health care industry and is being measured by governing institutions to improve healthcare delivery. High levels of patient satisfaction are associated with better outcomes, and waiting time is directly related to patient dissatisfaction. In an ideal world, no patient would ever wait to see his or her doctor, thus erasing the necessity of a waiting room. However, in today’s ever increasingly busy medical clinic, waiting time is an unavoidable reality, so identifying ways to lessen wait time would be ideal, considering the many negative consequences of increased wait time [1, 2]. Anecdotally speaking, the majority of patients’ negative feedback includes comments on wait time [3, 4]. Studies too have shown long wait times are associated with low patient satisfaction scores [5, 6]. It has been established that patients who are satisfied with their care are more likely to adhere to treatment [7]. Low patient satisfaction affects treatment compliance, including return visit rates [1, 8].

### Available tools

Many medical practices use a “trial and error” method to determine the optimal clinical arrangement, which often results in failed attempts and settling for a “good enough” option. Operations research techniques such as queuing theory, optimization techniques, and discrete-event simulations have been developed to understand and improve outpatient clinic performance [9–11]. Mathematical models based on queuing theory and linear and non-linear programming techniques often suffer from unrealistic assumptions which oversimplify the actual system [12].

### Simulation models

Discrete-event simulation models have been used in modeling complex outpatient clinic situations because they can depict the actual clinic without unrealistic assumptions while also accurately capturing random variations of the clinic [13]. Simulation models have been used successfully for outpatient clinics to streamline patient flows and to optimize resource allocations and utilization [14, 15]. In healthcare systems, simulation models can explore various performance measures such as patients’ wait time, resource utilization, resource allocation, system capacity, and appointment scheduling without altering the actual system [10, 16]. As opposed to continuous simulation, discrete-event simulation (DES) can test systems that change states at discrete epochs over time [17]. In this paper, we collected patient wait times at time epochs when they moved from one location to another location within the clinic. Since these movements take place at discrete time points, DES is a natural choice to our work.

### Appointment scheduling system

Outpatient clinics are often driven by appointment scheduling systems that infrequently accommodate same-day appointments. It is critical to have efficient appointment schedules in order to deliver high quality of service [18]. Several methods have been proposed to design appointment schedules [18–21]. Researchers tested the idea of scheduling multiple patients per block and found that it reduced patient wait time and doctors’ idle time in some cases and increased in some other cases [22, 23]. The variability in service times of providers, nature of treatment, and type of patients affect the performance of appointment systems [24]. For this reason, any generalized appointment scheduling rule will not necessarily improve the performance of a clinic [21, 25, 26].

### Resource allocation and utilization

Rising costs make it difficult for the health care services to recruit more staff. Hence, identifying optimum number of staff is pertinent to match the demand while maximizing staff utilization and minimizing patients’ wait time. The healthcare community has used simulation models effectively to identify optimal number of resources [27, 28]. Studying the changes to clinic resources and appointment scheduling policy simultaneously was found to be very effective in reducing patient wait times without compromising the resources’ utilization [29, 30].

### Simulation and optimization techniques

In order to automate the process of identifying optimal values for the simulation parameters such as the number of resources and time between appointments, suitable and efficient optimization techniques are needed to carry out several “what-if” scenarios with varying parameter values. Combination of optimization techniques and simulation techniques is found to be useful and efficient in simulating healthcare systems [31–33].

### The problem and proposed solution

The University of Arkansas for Medical Sciences (UAMS) recently opened a women’s health and internal medicine outpatient clinic in a high-growth area of Little Rock, Arkansas, with the mission to recruit and serve residents of this area through this conveniently located clinic, referred as the West Little Rock (WLR) clinic. To encourage patient retention at the new clinic, UAMS placed a high priority on decreasing patient wait times in the clinic. The new clinic very soon started facing long patient wait time around noon and late in the afternoon. To tackle this problem, the UAMS OB/GYN administration had tested various, new system-restructuring ideas such as changing the appointment scheduling system and the number of nurses, often with little or no success.

Witnessing little progress despite these time-intensive interventions, we proposed to develop a simulation model of the clinic to test new ideas to reduce patients’ wait time. By using the patient tracker (PT) data to fit probability distributions of service times of staff, the simulation model was fine-tuned further to test proposed changes to the system. We then validated the model by comparing the simulated wait times with the actual wait times calculated from the PT data.

In this work, we combined the validated simulation model and optimization technique to carry out “what-if” scenarios to identify an optimal appointment system and an optimal number of nurses. The results of these scenarios are discussed in detail through tables and time series graphs.

To further the discussion, the proceeding sections explore the layout and characteristics of the study site, the data collection process, the simulation software and study-specific development, the results, discussions, conclusions, and future research plan.

## Methods

### Clinic description

#### Patients

**Fig. 1:**
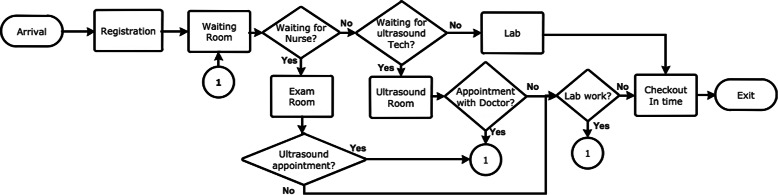
Flow of OB/GYN patients in the clinic

The WLR clinic handles three classes of patients: normal obstetric care (OB), complete gynecological service (GYN), and internal medicine service (IM). In addition, a fourth class of OB patients is handled by the clinic who had only ultrasound (US) appointments. Each of the first three classes involves two types of patients: new (NOB, NGYN, and NIM) and return (ROB, RGYN, and RIM) patients. Since the UAMS OB/GYN department elected to carry out “what-if” scenarios, this paper focuses only on the OB and GYN patient classes. However, all patients of the clinic share the common lab for blood work; therefore, the IM clinic is implemented in the simulation model and its details are omitted in this work. A typical flow of OB/GYN patients in the clinic is shown in Fig. 1.

#### Clinic layout

**Fig. 2:**
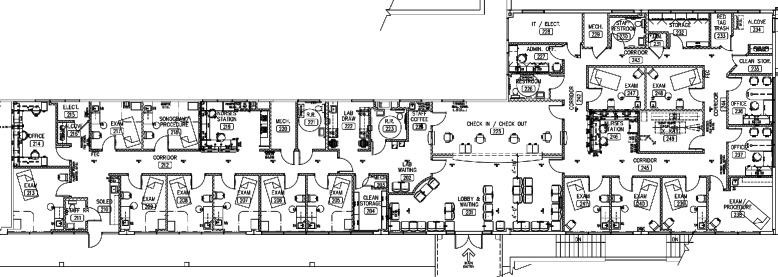
Clinic layout

Figure 2 shows the layout of the WLR clinic. The left side of the clinic is dedicated to OB/GYN patients and services, while the right side of the clinic serves IM patients. The front desk (225), waiting room (201), and one single capacity lab room (222) are the only locations in the clinic used by both OB/GYN and IM patients. In addition to these locations, the OB/GYN clinic has seven single capacity exam rooms (205 – 209, 213, and 217), one doctors’ office (214), one single capacity US room (218), and one nurses’ station (219).

#### Types of staff

There are five OB/GYN doctors, and they are referred to as Dr. A through Dr. E. There is one registered nurse (RN), two licensed practical nurses (LPNs), one medical assistant (MA), one patient representative (PR), two access coordinators (ACs), and one ultrasound technician (UST). The RN escorts patients from waiting room to exam rooms, checks vital signs, and chaperones GYN patients in the exam room if needed by the doctors. The duties of the two LPNs are the same as the RN. The MA conducts lab work for both OB/GYN and IM clinics. If available, she also chaperones GYN patients in the exam rooms and escorts patients from waiting room to exam room, but either an RN or LPN checks the vital signs of these patients. The PR assists with check-in when needed, balances the cash collection/batching, counsels patients on financial aspects, and also obtains all pre-authorizations for both clinics. The ACs manage the front desk: one for check-in and another for check-out. The UST performs diagnostic medical sonographic procedures. The working schedules of staff are tabulated in Table 1. On any working day, there are two physicians scheduled except on Wednesday when two 0.5 physicians (each worked a half day) are scheduled.

**Table 1:** Working schedules of staff

	Mon	Tue	Wed	Thu	Fri
Dr. A		AM & PM		PM	AM & PM
Dr. B				AM & PM	AM & PM
Dr. C		AM & PM	PM		
Dr. D	AM & PM			AM	
Dr. E	AM & PM		PM		
PR	AM & PM	AM & PM	AM & PM	AM & PM	AM & PM
RN	AM & PM	AM & PM	AM & PM	AM & PM	AM & PM
LPN	AM & PM	AM & PM	AM & PM	AM & PM	AM & PM
MA	AM & PM	AM & PM	AM & PM	AM & PM	AM & PM
AC	AM & PM	AM & PM	AM & PM	AM & PM	AM & PM
UST	AM	AM		AM	AM

AM denotes the morning session and PM denotes the afternoon session

#### Appointment (scheduling) template of providers

In total, 16 patients in the morning and 12 patients in the afternoon are scheduled for each provider. Out of 28, five and two are new patients scheduled in the morning and afternoon sessions, respectively, as per the clinic director’s recommendation. Hence, 56 patients are scheduled on Monday, Tuesday, Thursday, and Friday, and 24 patients on Wednesday. The appointment scheduling template currently used for a provider is shown in Table 2. The appointment times denote the appointment times with the providers and not for the ultrasound (US) procedure. US appointments preceded the providers’ scheduled appointments and they are not considered in this study. The performance measures *WT*,*TWT*, and *TST* (refer Table 5 for details) do not include US procedures.

**Table 2:** Appointment scheduling template

Morning Session		Afternoon Session	
Time	Procedure	Time	Procedure
7:45	ROB & NGYN	12:45	ROB & NGYN
8:00	RGYN	13:00	RGYN
8:15	NOB & ROB	13:15	NOB & ROB
8:30	RGYN	13:30	ROB & RGYN
8:45	NGYN	13:45	ROB
9:00	ROB	14:00	ROB
9:15	NOB	14:15	ROB
9:30	RGYN	14:30	SDA
9:45	ROB	14:45	SDA
10:00	RGYN		
10:15	NOB		
10:30	RGYN		
10:45	ROB		
11:00	ROB		

SDA stands for Same Day Appointment: ROB or RGYN - 50 % - 50 %

### Data

#### Patient tracker

**Fig. 3:**
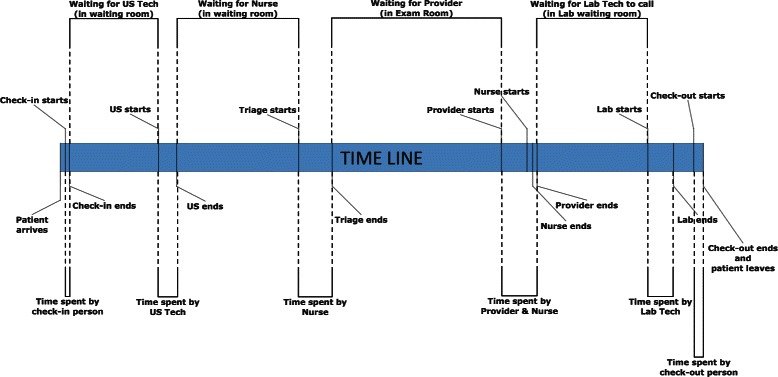
Milestones captured by staff using the PT software

Patient tracker (PT) is an in-house built, visualization and touch-screen software installed on all computers at various locations of the clinic, such as the front desk, exam rooms, US room, and labs. It was originally intended to record available exam rooms and assign patients to those rooms from the waiting room. The software was modified with additional buttons to record timestamps (milestones) whenever the staff interacted with patients at various locations. The staff were trained to use these added buttons. The milestones that were captured by the software are shown in Fig. 3.

Six months of data were collected for this study. During this period, the timestamps of more than 300 patients were collected for each staff listed in Table 1. From the data, the following details were gathered: (1) Show up percentages of patients, (2) service times of staff (amount of time a staff spent with a patient), (3) wait time of patients at various locations, (4) total wait times of each patient in the clinic, and (5) total spent time of each patient in the clinic. The total wait time of a patient is calculated by adding her wait times at various locations of the clinic. *TWT* is the average of total wait times. The total spent time of a patient is calculated by finding the difference in her check-in and check-out times. *TST* is the average of total spent times. The first two information are used in building the simulation model, and the last three are used to validate the simulation model.

#### Arrival times of patients

During UST working days (refer Table 1), US appointments were scheduled for every 30 min starting from 8 am to 11 am. US appointments preceded the providers’ scheduled appointments. The arrival times of patients, who had ultrasound procedure appointments, denote the times they come back to the waiting room after ultrasound procedure. For patients who did not have ultrasound procedure appointment, their arrival times denote the times they arrived at the clinic. The distribution of time differences between appointment time with the provider and the arrival times of patients was found to be normally distributed with mean 0 min and standard deviation (*SD*) 7 min. In addition, new patients were instructed to come 15 min early to complete the paperwork. We used this information in the simulation model while generating patients according to the appointment template.

#### Show up percentages

Show up percentages of patients of the five OB/GYN doctors are tabulated in Table 3. These percentages are the ratio of the actual number of arrived patients calculated from the PT data to the total number of scheduled patients calculated from the appointment template.

**Table 3:** Show up percentages of patients

		Mon	Tue	Wed	Thu	Fri
Dr. A	New Pt (%)		80		70	90
	Ret Pt (%)		75		75	78
Dr. B	New Pt (%)				71	75
	Ret Pt (%)				50	66
Dr. C	New Pt (%)		90	100		
	Ret Pt (%)		83	94		
*Dr. D	New Pt (%)	93			55	
	Ret Pt (%)	80			100	
Dr. E	New Pt (%)	56			100	
	Ret Pt (%)	80		95		

#### Service times of staff

**Fig. 4:**
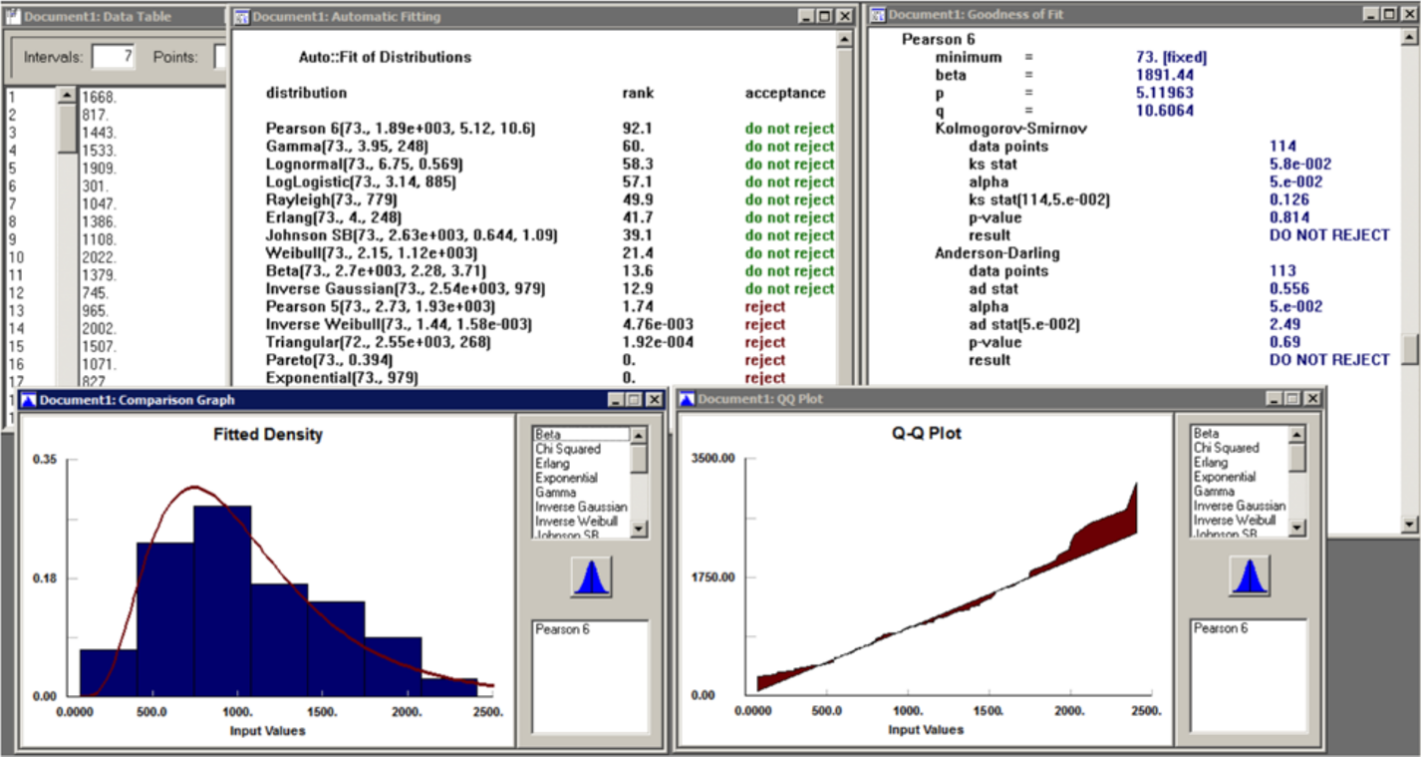
Sample output of Stat::Fit

Extracted service times (in minutes) of all staff from the PT data were used to identify suitable probability distributions using the Stat:Fit software [34]. Stat:Fit includes 32 probability distributions. Details about the notations and parameters of these distributions can be found in the user’s manual of the software. Stat::Fit ranks the best fit according to *p* values of two hypothesis tests: Kolmogorov-Smirnov (KS) and Anderson-Darling (AD). A screenshot of a fitted distributions is shown in Fig. 4. Though more than one distribution was identified by Stat::Fit as good fit for each staff member’s service time, they produced statistically significantly the same simulation results. Thus, in the simulation model, we used the top-ranked fitted distributions that are tabulated in Table 4 along with *p* values.

**Table 4:** Fitted service time distributions of staff

		Fitted distribution	KS *p* value	AD *p* value
US tech	New Pt	Triangular (10,15,20)	.427	.318
	Ret Pt	Triangular (10,15,20)	.631	.434
Check-in staff	New Pt	Weibull (5,1.96,700) / 60.0	.685	.677
	Ret Pt	Beta (5,2.1e + 003,1.96,14.6) / 60.0	.884	.751
Nurse	New Pt	LogLogistic (100,2.92,417) / 60.0	.934	.976
	Ret Pt	Pearson6 (51,1.32e+003,2.31,9.93)/60.0	.821	.81
Dr. A	New Pt	Pearson6 (73,1.89e + 003,5.12,10.6) / 60.0	.814	.69
	Ret Pt	Pearson6 (8,1.99e + 003,3.14,9.16) / 60.0	.884	.834
Dr. B	New Pt	Gamma (30,3.02,391) / 60.0	.995	.979
	Ret Pt	LogLogistic (2,2.57,818) / 60.0	.996	.913
Dr. C	New Pt	Weibull (185,1.71,904) / 60.0	.998	.999
	Ret Pt	LogLogistic (36,2.85,555) / 60.0	.791	.659
Dr. D	New Pt	Erlang (61,5,188) / 60.0	.941	.777
	Ret Pt	Gamma (20,2.38,285) / 60.0	.52	.513
Dr. E	New Pt	LogLogistic (121,3.17,741) / 60.0	.967	.944
	Ret Pt	Pearson6 (22,461,8.31,6.62) / 60.0	.641	.664
Lab tech	New Pt	Pearson6 (1,4.95e + 003,1.57,18.5) / 60.0	.911	.992
	Ret Pt	Lognormal (9,5.87,.995) / 60.0	.221	.172
Check-out staff	New Pt	Triangular (1,2,3)	.628	.547
	Ret Pt	Triangular (1,2,3)	.846	.652

#### Percentage of patients who had lab work

From the PT data, we computed the percentages of patients who had lab work. They were 100 % for NOB, 30 % for ROB, 60 % for NGYN, and 60 % for RGYN. In addition, 100 % of NIM and 30 % of RIM had lab work. Since the lab is used by both OB/GYN and IM clinics, this information is used in the simulation model.

*Ethics statement.* This was a simulation and modeling quality improvement (QI) project and generic times spent in different locations in clinic were computed from an existing template and no identifiable patient specific information was used in any part of the project. It was determined that this does not meet the definition of human subject research that would be subject to UAMS IRB oversight. It is not required under IRB Policy 1.4. The authors can provide all the generic data incorporated in the project with no specific information other than data related to time spent in each location averaged over many entries be made available to the journal or other researchers on request within guidelines of the institution.

### Simulation model

#### MedModel

**Fig. 5:**
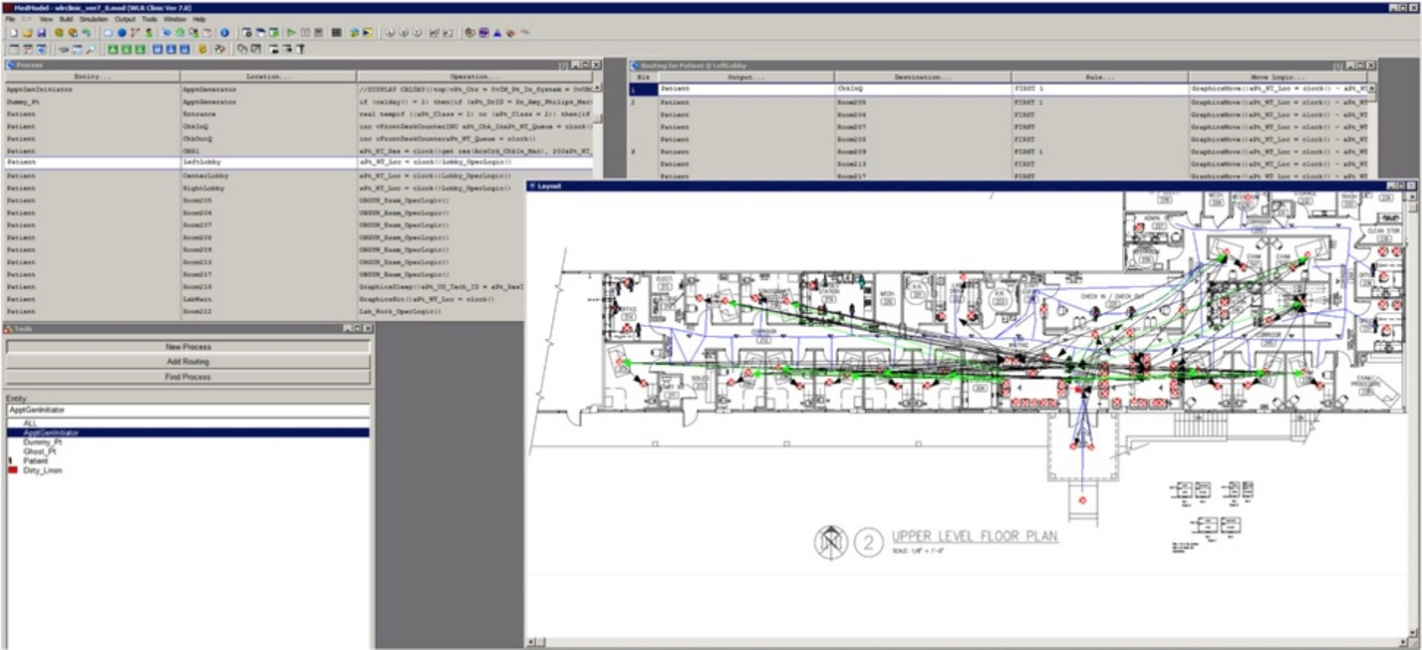
Processes in MedModel

**Fig. 6:**
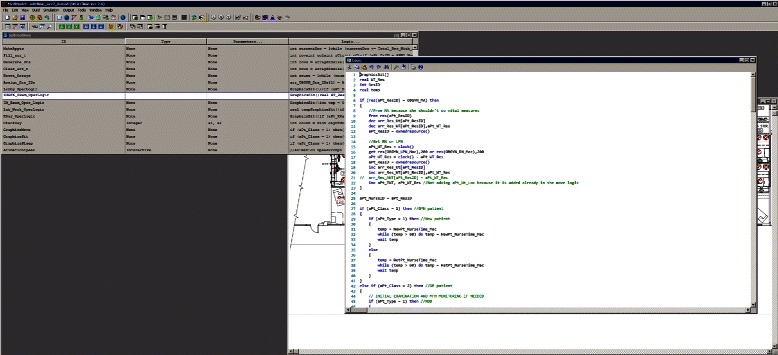
Subroutines in MedModel

**Fig. 7:**
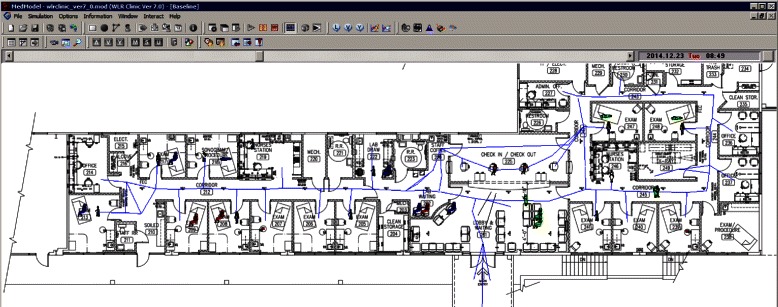
Running simulation in MedModel

We used MedModel [35], a discrete-event simulation software, to develop the simulation model for the clinic. The major steps to build a simulation in MedModel are sequenced logically: locations, entities (patients), resources (staff), arrivals, and processing. Most of the codings in processing steps define flows of patients through the clinic and how they are processed by the staff. Figure 5 is a screenshot of the model with process tables. Codings which are repeated in different processes for various parameters are modularized as subroutines. Figure 6 is a screenshot of the model with subroutines. MedModel’s visualization feature helps the developer to validate the flow of patients and staff in the clinic. Figure 7 is a screenshot of a running simulation in MedModel. MedModel’s scenario manager helps researchers carry out various “what-if” scenarios manually to identify optimal values for the clinic parameters.

#### Simulation code

Since there are more than 4000 lines of coding involved in developing the simulation model, we made these codes available in a simple text format at sites.google.com/a/uca.edu/rblenin/biomed.

#### Input parameters

We used the following information as input parameters for the simulation model: (1) Types and number of patients scheduled, (2) types and number of staff, (3) appointment templates of providers and UST, (4) show up percentages of patients, (5) arrival times of patients, (6) service times of staff, and (7) percentages of different types of patients who had lab work.

#### Output parameters

We measured the following output from the simulation model: (1) *WT*, (2) *TWT*, (3) *TST*, (4) utilization of staff and exam rooms, and (5) throughput. We then compared the measures (1) - (3) with the ones computed from the exact (Patient-Tracker) data to validate the developed simulation model. The validated model is then used to carry out what-if analyses. Since we measure wait times of patients at various locations (*WT*) and their overall wait times and spent times (*TWT* and *TST*), we refer these simulation outputs as micro (for local wait times) and macro (for overall wait times and spent times) simulation analyses. We note that, in this work, the terms “micro” and “macro” are not used to represent short-term and long-term simulation durations, respectively.

#### SimRunner

We used SimRunner, a decision support toolbox of MedModel, to find the optimal solution of the optimization problem, which is detailed later in this paper. SimRunner takes the optimization problem as its input and runs the validated simulation model that is built using MedModel to find the optimal solution using both genetic [36] and evolutionary algorithms [37]. These algorithms help to explore specific values of contraint variables to achive the optimal solution which in turn reduces the computational complexity of the optimization algorithm.

#### Notations

Notations used for the performance measures of the clinic are tabulated in Table 5. We note that the throughput, ${\mathcal {T}}$, is the total number of checked out patients by 5 pm if the physician is either scheduled for the whole day or for the afternoon session or the total number of checked out patients by 1 pm if the physician is scheduled for the morning session. These time limits were recommended by the clinic director.

**Table 5:** Notations for the performance measures of the clinic

Acronym	Description
*WT*	Average wait time of a patient for the doctor in the exam room (in min)
*TWT*	Average total wait time of a patient for the resources at various locations of the clinic (in minutes)
*TST*	Average total time of a patient from check-in to check-out (in minutes)
${\mathcal {T}}$	Throughput - the total number of patients seen by a physician per day

#### Model validation

To validate the simulation model, the *TWT* (see Table 5 for acronym descriptions) computed from the simulation results, after running 30 replications, were compared in Table 6 with the actual *TWT* extracted from the PT data. The comparison showed no statistical difference between the simulation results and actual data. Also, the comparison of *WT* and *TST* showed no statistical difference between the actual and simulation results. We used 30 replications because a sample size of 30 or more is needed to guarantee the condition that the sample mean approximately follows a normal distribution, according to the Central Limit Theorem for sample means. This condition is needed to check whether or not the difference between the actual and simulation results is statistically significant. These replications were simulated for each working day starting at 7:30 am and ending at 5 pm. The “what-if” analyses in the next section were carried out using the validated simulation model.

**Table 6:** Comparison of actual and simulation results

		*TWT*		
		Actual data	Simulation result	*p* value
Mon	Dr. D	24	22.34	0.371
	Dr. E	21	18.99	0.267
Tue	Dr. A	35	37.83	0.539
	Dr. C	33	34.11	0.761
Thu	Dr. A	31	33.37	0.542
	Dr. B	36	33.16	0.495
	Dr. D	29	27.14	0.569
Fri	Dr. A	21	22.14	0.596
	Dr. B	24	25.37	0.118

### What-if analyses

#### Varying number of staff

As the physicians are the most expensive staff, we carried out “what-if” analyses by varying the number of RNs, LPNs, and MAs from 1 to 4. Other staff were represented the same as in the validated model. We refer to the validated model as the *baseline* model.

#### Modifying appointment template

The changes to the appointment template such as reducing the number of double appointments or keeping the double appointments but moving them to different time slots did not improve the wait time of patients. We observed that the double appointments caused more people to wait in the waiting rooms resulting in increased *WT* which in turn resulted in increased *TWT* and *TST*. Hence, we decided to modify the existing appointment template in Table 2 by splitting all double (two in one time slot) appointments into two single appointments in different time slots by creating two new time slots in the AM session and three slots in the PM session as shown in Table 7. This type of changes to the appointment template was found to be effective as pointed out by Ho and Lau [23]. We used this modified template to carry out “what-if” analyses to identify the optimal duration of each appointment slot by varying the duration *t*_*am*_ from 10 min to 16 min for the morning appointments and by varying the duration *t*_*pm*_ from 10 min to 21 min for the afternoon appointments with the constraint that all patients are checked out by 5 pm or earlier. The minimum value of *t*_*am*_ and *t*_*pm*_ is set to be 10 min as per the clinic director’s recommendation. The maximum value for *t*_*am*_ is set to 16 min in order to schedule all 16 patients in the morning by 12 noon whereas for *t*_*pm*_, the maximum value is set to 21 min in order to schedule all 12 patients before 5 pm. We tested two different durations for morning and afternoon sessions in order to meet varying demands of these sessions which may potentially lead to overtime. This type of policies are referred as overload rules and rule delay in the literature [26].

**Table 7:** Modified appointment scheduling template

Morning session	Afternoon session
(starting at 7:45 am)	(starting at 12:45 pm)
ROB	ROB
NGYN	NGYN
RGYN	RGYN
NOB	NOB
ROB	ROB
RGYN	ROB
NGYN	RGYN
ROB	ROB
NOB	ROB
RGYN	ROB
ROB	SDA
RGYN	SDA
NOB	
RGYN	
ROB	
ROB	

#### Optimization problem

We analyzed the following optimization problem:

(1) $$\begin{array}{@{}rcl@{}}  & & \hspace*{1in} \text{Minimize \textit{TWT}} \end{array} $$

subject to the constraints

(2) $$\begin{array}{@{}rcl@{}} & & 1\leq x_{i} \leq 4,\quad i = 1,2,3,  \end{array} $$

(3) $$\begin{array}{@{}rcl@{}} & & 10 \leq t_{am} \leq 16,  \end{array} $$

(4) $$\begin{array}{@{}rcl@{}} & & 10 \leq t_{pm} \leq 21, \text{~~and}  \end{array} $$

(5) $$\begin{array}{@{}rcl@{}} & & \text{all patients are checked out by 5pm or earlier.}  \end{array} $$

Here *x*_1_ denotes the number of LPNs, *x*_2_ denotes the number of RNs, *x*_3_ denotes the number of MAs, and *t*_*am*_ and *t*_*pm*_ denote the durations of each slot of the appointment template in Table 7 for morning and after sessions, respectively. In the constraint (2), we set the minimum number to be one for each type of nurses to make sure that at least one nurse of each type is available for the clinic model as they render different types of services.

We note that the number of nurses is independent of the slot durations. This is because the amount of time a nurse spends with a patient is obtained from the fitted probability distribution (refer Table 4) and it does not depend on the duration of the time slot. The shorter time slots lead to early arrival of patients which in turn increases the wait time of patients in different locations of the clinic due to heavy traffic, especially in the morning sessions and early afternoon sessions. Hence, it is necessary to increase the number of nurses to ease the traffic. However, on the other hand, the longer time slots lead to light traffic but less throughput. Hence, it is pertinent to identify the optimal values for the parameters in the constraints (2-4) while satisfying the constraint (5) and minimizing the objective function in (1).

**Fig. 8:**
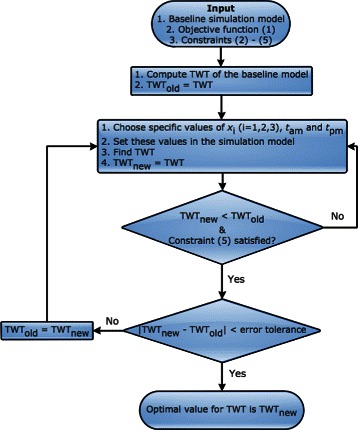
Schematic diagram of functionality of SimRunner

**Fig. 9:**
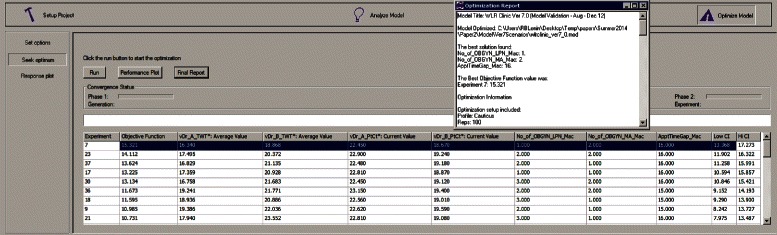
Sample output of SimRunner

The validated simulation model is modified with testing parameters based on the constraints (2) - (5). We configured SimRunner for the above optimization problem, which in turn ran the validated model for all possible parameter values in the constraints, using a sophisticated algorithm, until it found the solution that globally minimized the objective function (1). A schematic diagram of the functionality of SimRunner is shown in Fig. 8. A screenshot of an output of SimRunner is shown in Fig. 9. The optimal solution that minimized the objective function (1) is *x*_1_=2,*x*_2_=1, and *x*_3_=2,*t*_*am*_=16, and *t*_*pm*_=19. This means, the optimal solution is achieved by adding one more MA and keeping the morning appointments 16 min apart and afternoon appointments 19 min apart. We refer the model with these parameter values as the *best* model.

## Results

Being a light day in terms of the number of patients scheduled, Wednesday is omitted in the “what-if” analyses. Using the optimal parameter values, we ran the simulation with 30 replications for all other working days.

### Wait time in the waiting room

**Fig. 10:**
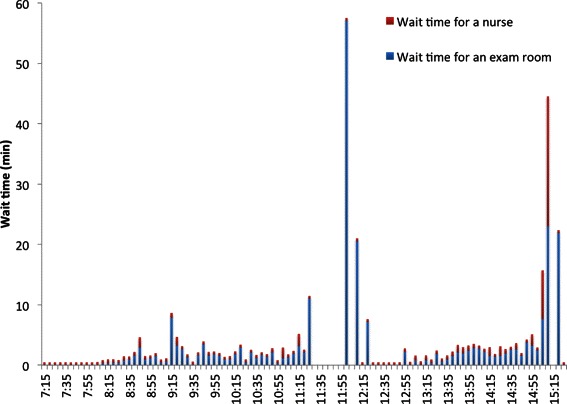
Average wait time of patients in the waiting room for nurses to escort them to exam rooms in the baseline scenario

**Fig. 11:**
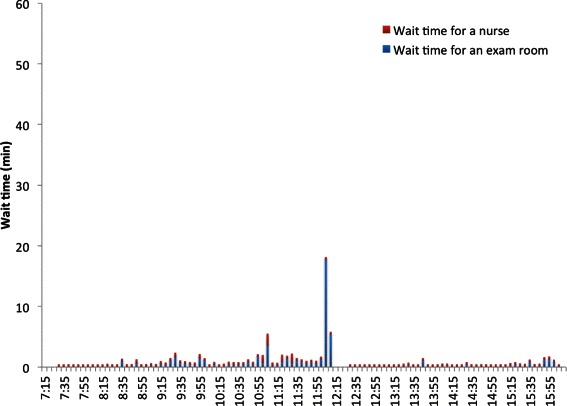
Average wait time of patients in the waiting room for nurses to escort them to exam rooms in the best scenario

Figure 10 shows the average wait time of patients of all providers in the waiting room for nurses to escort them to exam rooms for the baseline scenario. The corresponding results for the best scenario are shown in Fig. 11.

### Utilization of staff and exam rooms

We define the average utilization of a staff member

$${} {\fontsize{8.4pt}{9.6pt}\selectfont{\begin{aligned} = \frac{\text{total face-to-face contact time of the staff with patients per day}}{\text{total scheduled work hours of the staff per day}}. \end{aligned}}} $$

It does not factor in other duties of staff, such as charting and attending phone calls. Similarly, the average utilization of an exam room

$$\begin{array}{@{}rcl@{}} & = & \frac{\text{total time patients stayed in the exam room per day}}{\text{total scheduled work hours of the clinic per day}}. \end{array} $$

**Fig. 12:**
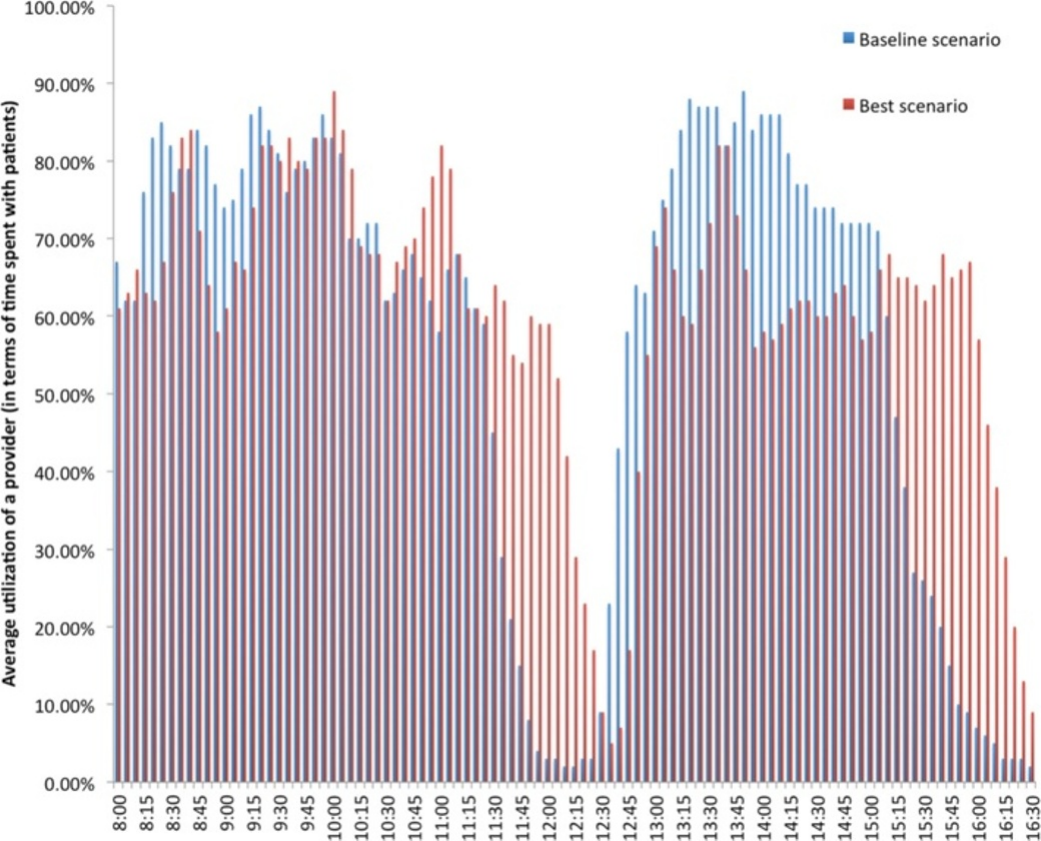
Utilization of a provider

**Fig. 13:**
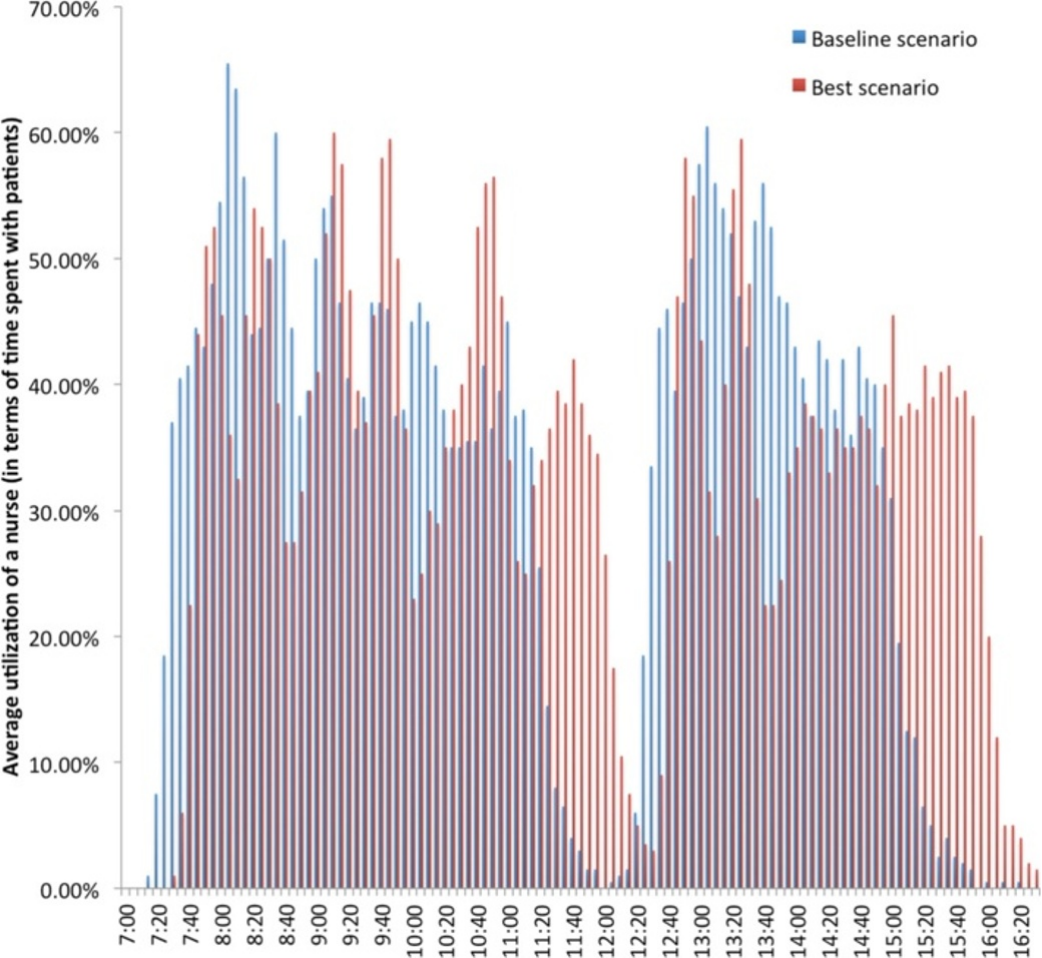
Utilization of a nurse

**Fig. 14:**
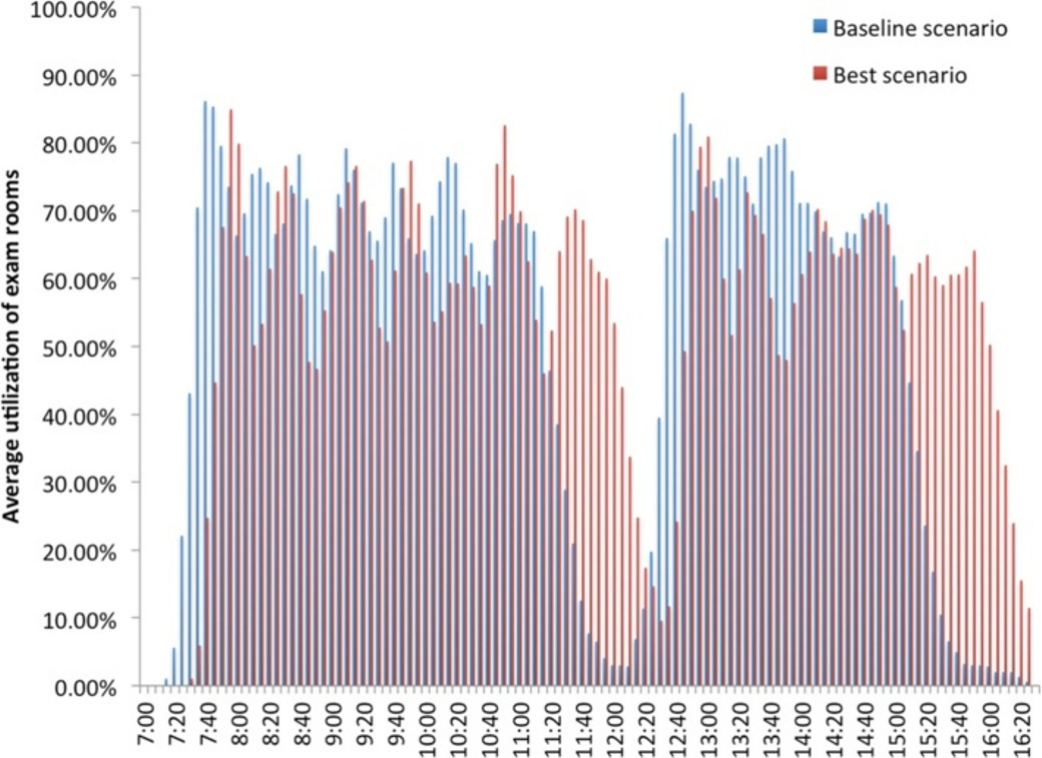
Utilization of an exam room

In Table 8, the average utilizations of providers, a nurse, and an exam room are tabulated. The utilization of providers, nurses, and exam rooms throughout a typical working day of the clinic are depicted in Figs. 12, 13 and 14.

**Table 8:** Average utilization of staff and exam rooms

		Average utilization (%)	
		*Baseline*	*Best*
Mon	Dr. D	72.20	69.71
	Dr. E	61.47	60.41
	Nurse	34.49	36.46
	Exam room	54.56	58.94
Tue	Dr. A	69.08	69.17
	Dr. C	73.90	74.33
	Nurse	34.27	37.06
	Exam room	56.5	58.95
Thu	Dr. A	60.50	51.55
	Dr. B	62.16	57.41
	Dr. D	71.27	68.21
	Nurse	30.72	31.96
	Exam room	46.33	47.45
Fri	Dr. A	73.48	71.26
	Dr. B	74.39	70.68
	Nurse	32.73	34.33
	Exam room	57.61	59.4

### Average wait times *WT*,*TWT*,*TST*, and throughput $\boldmath {{\mathcal {T}}}$

In Tables 9 and 10, the performance measures *WT*,*TWT*,*TST*, and ${\mathcal {T}}$ of the clinic corresponding to the baseline and best scenarios are compared.

**Table 9:** Comparison of *Baseline* and *Best* scenarios

		*WT*				*TWT*			
		*Baseline*	*Best*	Improvement (%)	*p*	*Baseline*	*Best*	Improvement (%)	*p*
Mon	Dr. D	10.36	5.46	47.30	<.001	22.34	14.11	36.84	<.001
	Dr. E	6.38	3.75	41.22	<.001	18.99	12.42	34.60	<.001
Tue	Dr. A	9.84	5.92	39.84	<.001	37.83	28.60	24.40	.005
	Dr. C	8.32	5.46	34.38	<.001	34.11	27.07	20.64	.008
Thu	Dr. A	8.02	3.72	53.62	<.001	33.37	17.34	48.04	<.001
	Dr. B	10.38	5.68	45.28	.005	33.16	22.05	33.50	<.001
	Dr. D	8.31	5.27	36.58	<.001	27.74	21.89	21.09	.013
Fri	Dr. A	11.37	7.96	29.99	<.001	22.14	16.48	25.56	<.001
	Dr. B	12.32	8.58	30.36	<.001	25.37	18.24	28.10	<.001
Overall		9.48	5.76	39.84	<.001	28.34	19.80	30.31	<.001

**Table 10:** Comparison of *Baseline* and *Best* scenarios

		*TST*				${\mathcal {T}}$	
		*Baseline*	*Best*	Improvement (%)	*p*	*Baseline*	*Best*
Mon	Dr. D	55.00	45.50	17.27	<.001	24	24
	Dr. E	51.37	44.20	13.96	<.001	21	21
Tue	Dr. A	72.09	63.80	11.50	.019	22	22
	Dr. C	66.66	59.69	10.46	.015	24	24
Thu	Dr. A	65.61	50.10	23.64	<.001	9	9
	Dr. B	69.85	57.04	18.34	<.001	16	16
	Dr. D	59.59	52.98	11.09	<.001	14	14
Fri	Dr. A	55.80	46.30	17.03	<.001	23	23
	Dr. B	62.95	54.90	12.79	<.001	19	19
Overall		62.10	52.72	15.12	<.001		

**Fig. 15:**
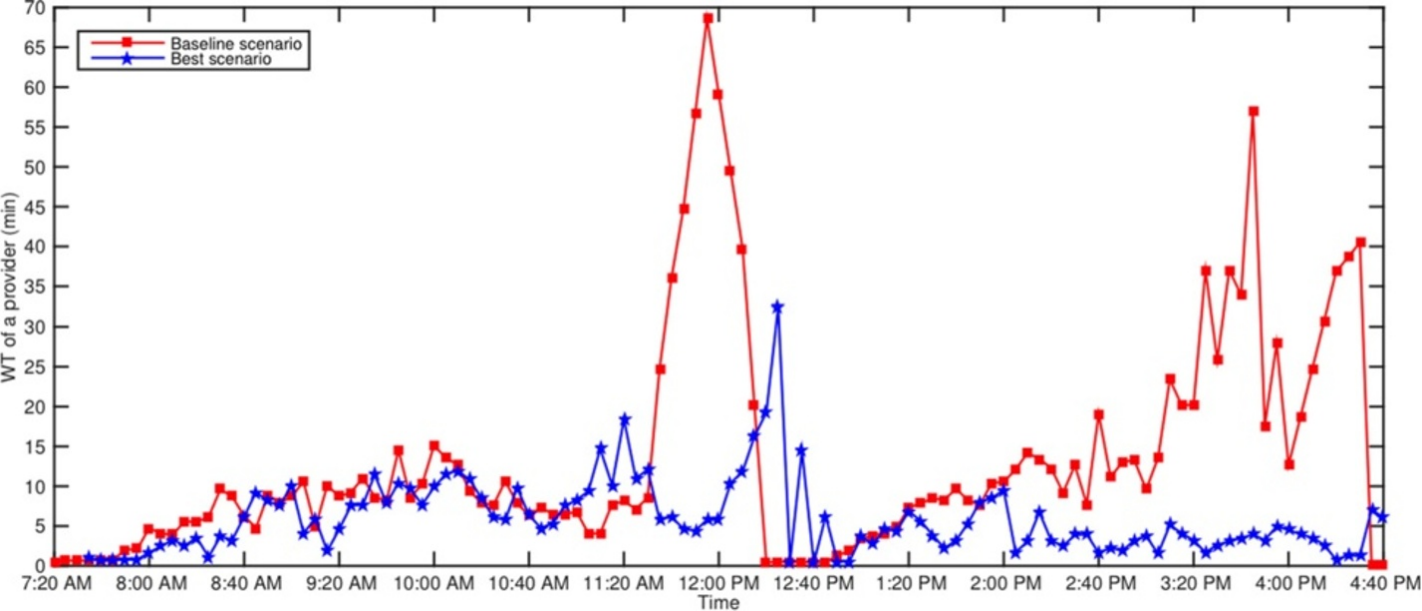
*WT* of Dr. C’s patients on a Tuesday

**Fig. 16:**
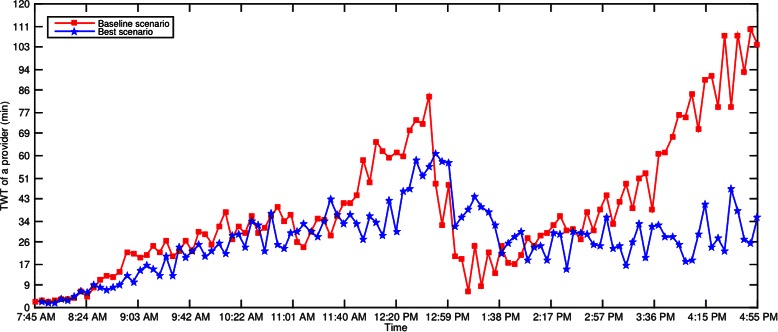
*TWT* of Dr. C’s patients on a Tuesday

The time series graphs of *WT* and *TWT* of Dr. C’s patients on a Tuesday are shown in Figs. 15 and 16. Similar improvements were observed across all other days for other providers and hence their graphs are omitted in the paper for brevity.

## Discussions

### Justification for modifying the appointment template

A patient waits in the waiting room until a nurse escorts her to an exam room if an exam room is available. Wait time of all patients of all providers in the waiting room due to unavailability of exam rooms and due to unavailability of nurses were computed in the simulation model. The average values of these wait times are plotted in Fig. 10. It is clear from Fig. 10 that throughout the day, the major delay in moving patients from the waiting room to the exam rooms is caused by the unavailability of the exam rooms rather than by the unavailability of nurses. This proves the fact that this delay cannot be avoided by adding more nurses. Since reducing the number of scheduled patients or adding more exam rooms is not cost effective, the other feasible option is to make changes to the appointment scheduling template without reducing the number of scheduled patients. Hence, we decided to include the constraints (3) and (4) in the optimization problem (1) besides modifying the appointment template by breaking all double-appointment slots into two single-appointment slots by creating new slots as shown in Table 7.

In Fig. 10, corresponding to the baseline scenario, the two major peaks in wait time occur around 12 noon and around 3 pm. The first peak is due to unavailability of all exam rooms between 11:25 am and 12 noon as no patients were moved to the exam rooms. The second peak is due to ripple effect of wait times caused by previous patients who were scheduled in the early afternoon session. The second peak starts melting down as there are no more new arrival of patients after 2:45 pm.

Though the delay in moving patients to the exam rooms is still mainly due to unavailability of exam rooms in the best scenario as shown in Fig. 11, it is reduced substantially when compared to the baseline scenario (Fig. 10). In addition, the best scenario removed all major delays (e.g. more than 55 min around 12 pm) of the the baseline scenario. Moreover, between 11:30 am and 12 pm, in the baseline scenario, all exam rooms were occupied and no patients were moved from the waiting room to exam rooms during this time. This bottleneck is removed in the best scenario.

### Shift in the utilization of staff and exam rooms

The variations in the utilization of staff and exam rooms throughout a typical working day of the clinic are captured in Figs. 12, 13 and 14. The utilization of staff and exam rooms in the best scenario is shifted to the right when compared to the baseline scenario because of the changes made to the appointment template and the morning appointments were 16 min apart and afternoon appointments were 19 min apart in the best scenario as identified by the optimal solution.

### Significant reduction in wait times

The wait times *WT*,*TWT*, and *TST* have significantly decreased in the best scenario when compared to the baseline (Tables 9 and 10). Overall, the best scenario yielded 39.84 % (*p*<.001), 30.31 % (*p*<.001), and 15.12 % (*p*<.001) improvement in *WT*,*TWT*, and *TST*, respectively. These improvements were achieved without compromising the utilization of staff and exam rooms (Table 8), and the number of patients served by 5 pm as the remains the same for both baseline and best scenarios (Table 10). Consistent improvement in wait times is achieved in the best scenario throughout the day except at few time points (Figs. 15 and 16).

### Cost vs. reduction in wait time

Since both OB/GYN and IM clinics share the same lab and MAs are the only staff handling lab works, we achieved a significant reduction in the wait times *TWT* and *TST* of patients by adding one more MA besides making changes to the appointment template. According to the U.S. Bureau of Labor Statistics [38], the median annual salary of a MA is $29,610 which is the only additional foreseen cost to UAMS if recommended changes are made to the clinic.

## Conclusions

In this paper, we: 1) used MedModel, a discrete-event simulation software, to develop a simulation model for an OB/GYN clinic, 2) used the patient tracker data to fit appropriate probability distributions for service times of staff and to validate the model, and 3) carried out “what-if” analyses using the validated model to find the optimal solution of an optimization problem of minimizing the average wait times of patients in the clinic subject to the constraints of varying number of nurses and varying time between appointments of a modified appointment template. The existing appointment template was modified by breaking all double-appointment slots into two single-appointment slots by creating new slots.

The optimal solution was achieved by adding one more MA and keeping the morning appointments 16 min apart and afternoon appointments 19 min apart. Besides removing all peak wait times and bottlenecks around noon and late in the afternoon, the changes suggested by the optimal solution led to a significant reduction in the wait time of patients without compromising the utilization of the staff and exam rooms or throughput of patients. We achieved these improvements without affecting the actual clinic activities which the clinic management had difficulty achieving with many manual interventions. Moreover, these manual interventions did not reveal the fact that the unavailability of exam rooms was the main cause for the major delay in moving the patients from the waiting room to exam rooms. An extensive list of tables and figures were provided to describe the clinic operations, to describe the simulation tools, and to illustrate the improvements of all micro and macro performance measures of the clinic.

It has been observed that, on average, providers are occupied for at least 15 min more between 12:30 pm and 1 pm in the best scenario when compared to the baseline (Figs. 14 and 15) due to more appointment slots with 16 and 19 min apart for morning and after sessions, respectively. Similar, observation was made for nurses. This type of changes to the appointment template which lead to overtime of staff is referred as overload rules and rule delay in the literature. As part of the future plan, after consulting with the clinic management, staggered working schedules for nurses and providers will be proposed to address the overtime of staff.

The future plan is also to extend this model for the other two UAMS OB/GYN outpatient clinics to reduce patient wait times by identifying optimal number of resources for these clinics.
